# Evaluating potentially toxic element accumulation in crops near abandoned mine tailings in northwestern Mexico: a One Health perspective

**DOI:** 10.1007/s10661-025-14587-1

**Published:** 2025-09-20

**Authors:** Martha Camacho-Alcantar, Blanca González-Méndez, René Loredo-Portales, Jose Raul Romo-Leon, Francisco Molina-Freaner

**Affiliations:** 1 Posgrado en Biociencias, Universidad de Sonora, Hermosillo, Sonora México; 2 SECIHTI, Insurgentes Sur 1582, Ciudad de México, 03940 México; 3 Instituto de Geología, Estacion Regional del Noroeste, Universidad Nacional Autónoma de México, Hermosillo, Sonora México; 4 Departamento de Investigaciones Científicas y Tecnológicas, Universidad de Sonora, Hermosillo, Sonora México; 5 Departamento de Ecología de La Biodiversidad, Instituto de Ecología, Universidad Nacional Autónoma de México, Hermosillo, Sonora México

**Keywords:** Heavy metals, Metal accumulation, Rio Sonora, Soil pollution, Soil resilience

## Abstract

This study investigated the accumulation of potentially toxic elements (PTEs) in crops cultivated near abandoned mine tailings (MTs) in San Felipe de Jesús, northwestern Mexico, with the aim of evaluating their compliance with food safety regulations. A total of 26 soil and crop samples were analyzed using inductively coupled plasma mass spectrometry (ICP-MS). In soils, concentrations of lead (Pb), zinc (Zn), and cadmium (Cd) reached 367 mg kg⁻^1^, 781 mg kg⁻^1^, and 6.19 mg kg⁻^1^, respectively, and declined exponentially with distance from the MTs, consistent with wind-driven dispersion. Soils exhibited moderate to poor resilience, with 34.6% classified as poor based on pH, organic carbon, cation exchange capacity, iron oxides, and calcium oxides. In crops, several forage samples exceeded maximum tolerable levels (MTLs) for livestock: all barley and alfalfa samples surpassed the MTL for potassium (2.5% for cows and horses) and sulfur (0.4% for cows); some also exceeded the MTLs for calcium (1.5% for horses) and iron (500 mg kg⁻^1^ for horses). Among food crops, cadmium concentrations exceeded Codex Alimentarius limits in one peanut sample (0.15 mg kg⁻^1^; Limit: 0.1 mg kg⁻^1^), both pepper samples (0.08–0.09 mg kg⁻^1^; Limit: 0.05 mg kg⁻^1^), and chiltepin (0.10 mg kg⁻^1^; Limit: 0.05 mg kg⁻^1^). Lead concentrations in one pepper (0.09 mg kg⁻^1^) and chiltepin (0.10 mg kg⁻^1^) also surpassed the Codex Limit of 0.05 mg kg⁻^1^, whereas all maize samples remained below safety thresholds. These results highlight the health and environmental risks of legacy mining and support the need for phytostabilization and amendment-based remediation to reduce PTE mobility and crop uptake.

**Supplementary Information:**

The online version contains supplementary material available at 10.1007/s10661-025-14587-1.

## Introduction

The One Health approach highlights the interconnectedness of human, animal, and environmental health, recognizing that disturbances in one domain can trigger cascading effects in the others (Destoumieux-Garzón et al., 2018). This perspective is particularly relevant when examining environmental contaminants such as potentially toxic elements (PTEs) derived from mining activities, which degrade soil health, disrupt ecosystems, and ultimately pose risks to human well-being (Jota Baptista et al., 2024; Hou et al. 2025).

Soil health is fundamental to food production and ecosystem functioning, serving as the medium in which nearly all food crops grow (FAO and UNEP. 2021). However, it is increasingly threatened by contamination from trace metals, agrochemicals, and industrial waste (Vargas-Rojas et al., 2016). These pollutants enter soils via atmospheric deposition, water transport, and industrial discharges, accumulating in crops and thereby threatening both animal and human health (Hoffman et al. 2022). For example, spinach grown in soils containing 250 mg kg⁻^1^ Pb can accumulate over 10 mg kg⁻^1^ Pb in edible tissues, exceeding the Codex Alimentarius Limit of 0.3 mg kg⁻^1^ for leafy vegetables (Wang et al., 2021).

Among the primary sources of soil contamination is metal mining, especially through the storage and abandonment of mine waste (Macklin et al., 2023). Active and abandoned mines affect floodplains and river systems by dispersing contaminants through spills, wind erosion, and water flow (Kossoff et al., 2014). Mine tailings (MTs)—often left exposed and unmanaged for decades— may contain extremely high PTE concentrations, such as Pb > 2,000 mg kg⁻^1^ and As > 500 mg kg⁻^1^, far exceeding FAO/WHO soil safety thresholds (Pb 100 mg kg⁻^1^; As 20 mg kg⁻^1^) (Cross et al., 2017). Wind and water erosion can disperse these particles several kilometers from the source; in arid regions of the southwestern USA, Pb-rich dust from MTs has been detected up to 8 km away (Mendez & Maier, 2008). The extent of PTE dispersal depends on the topography, vegetation, and climate, all of which influence how far contaminants travel from mining sites into agricultural and residential zones (Candeias et al., 2014; Csavina et al., 2012).

In arid regions, wind-driven (eolian) transport dominates over fluvial transport, producing a typical pattern of high surface soil PTE concentrations near the source that decline exponentially with distance (Kim et al. 2012; Kim et al. 2014). For example, soils within 500 m of MTs contained 1,200–1,500 mg kg⁻^1^ Zn and 400–800 mg kg⁻^1^ Pb, dropping below 200 mg kg⁻^1^ Zn and 50 mg kg⁻^1^ Pb at distances > 5 km (Kim et al., 2014). Such contamination hotspots in nearby agricultural soils increase the risk of dietary exposure; maize grown in soils with 300 mg kg⁻^1^ Pb can accumulate 2–8 mg kg⁻^1^ Pb in kernels, while leafy vegetables may contain 10–20 mg kg⁻^1^ Pb (Liu et al., 2023). The degree of PTE accumulation in crops depends on soil properties, plant species, and the specific chemical forms of the PTEs involved. For instance, in mining-impacted areas of China, Cd concentrations in rice grains ranged from 0.12 to 0.48 mg kg⁻^1^, with soils containing 0.8–1.5 mg kg⁻^1^ Cd, frequently exceeding the Codex Alimentarius maximum Limit of 0.2 mg kg⁻^1^ for cereals (Wang et al., 2021). Monitoring PTE levels in crops is therefore essential to ensure food safety and to establish region-specific permissible concentrations.

Soil resilience—the ability of soil to reach a new equilibrium following disturbance—is critical to understanding its capacity to buffer contaminants (Song et al., 2022; Stigliani, 1996). Key properties such as pH, organic carbon content, and cation exchange capacity influence PTE mobility by regulating their retention or release in the soil matrix (Song et al., 2022). For example, in Mediterranean agricultural soils, increasing pH from 5.5 to 7.5 reduced soluble Cd by 60% (Song et al., 2022). However, mine waste contamination can undermine these buffering mechanisms, increasing the mobility and bioavailability of toxic elements (Zuñiga-Vázquez et al., 2023).

**Fig. 1:**
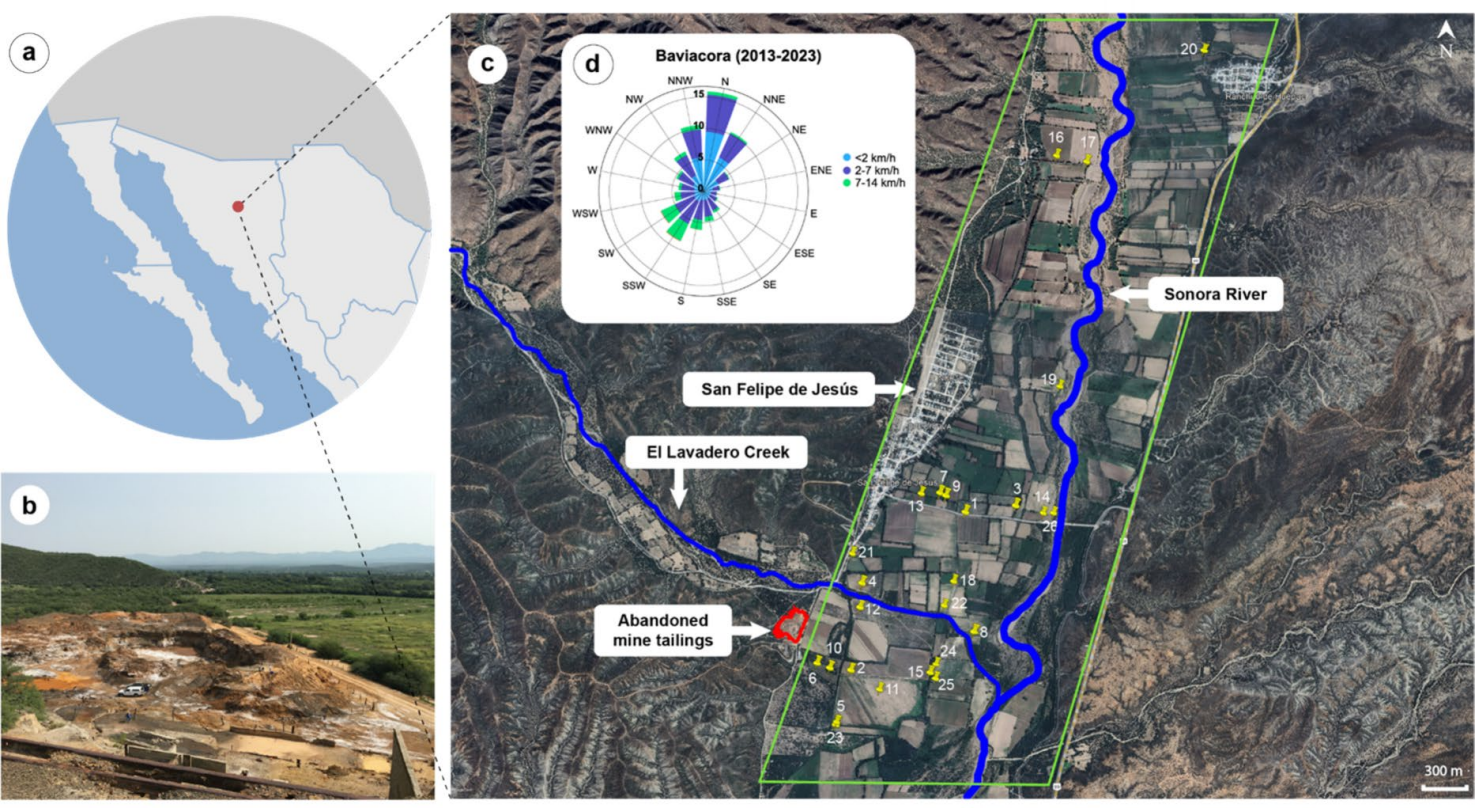
Study site. **a** Location of San Felipe de Jesús in northwestern Mexico. **b** View of the abandoned mine tailings close to agricultural fields in San Felipe de Jesús. **c** Google Earth view of the agricultural area of San Felipe, showing the location of the sampled fields, the town of San Felipe, Rio Sonora, Lavadero Stream and the abandoned mine tailings. **d** A wind rose of Baviacora (2013–2023), a nearby town, with data on wind speed and direction

The Sonora River basin in northwestern Mexico, is one of several regions affected by abandoned MTs, including the location near San Felipe de Jesús (Fig. 1b). During the dry season, wind erosion transports metal-rich dust to nearby agricultural soils, where crops are irrigated from wells, springs, or river water (Del Rio-Salas et al. 2019). Floodplain soils in the Sonora River vary in their ability to neutralize pollutants: soils rich in carbonate can immobilize PTEs, whereas carbonate-poor soils allow for greater mobility (Rivera-Uria et al. 2019; Ziegler-Rivera et al., 2020), which raises concerns about the persistence and long-term effects of pollution. However, the buffering capacity of agricultural soils in San Felipe—and their role in reducing PTEs mobility—remains poorly characterized.

Previous studies in San Felipe have documented soil As concentrations of 60–120 mg kg⁻^1^, Zn 900–1,400 mg kg⁻^1^, and Pb up to 1,800 mg kg⁻^1^ within 500 m of the tailings (Gonzalez-Mendez et al. 2022). These values exceed agricultural soil safety thresholds by factors of 3–10, and sequential extraction analyses indicate that 40–60% of these elements occur in bioavailable forms (Loredo-Portales et al., 2020; Morales-Pérez et al., 2021). Despite this, the extent of PTE accumulation in locally grown crops and the implications for food safety and public health have not been comprehensively evaluated.

To address this knowledge gap from a One Health perspective, this study investigates PTE accumulation in agricultural systems affected by abandoned mine tailings in San Felipe de Jesús. Specifically, we aim to: (a) quantify PTE concentrations in commonly cultivated crops (peanut, maize, pepper, chiltepin, alfalfa, barley, ryegrass, and *Sorghum halepense*) and the agricultural soils where they grow; (b) assess the relationship between soil physicochemical properties and PTE concentrations in plant tissues to identify potential predictors of contamination; (c) compare crop PTE concentrations to international food safety thresholds (e.g., Cd 0.1–0.2 mg kg⁻^1^ in cereals, Pb 0.3 mg kg⁻^1^ in vegetables; Codex Alimentarius) to identify contaminants of concern for human and animal health; and d) estimate a soil resilience index based on properties relevant to PTE retention, to assess the vulnerability of agricultural soils in the region.

This study applies the One Health framework, which integrates four components: a) an aspect of human health, b) an aspect of animal health, c) an aspect of environmental health and d) the intersections among these domains (Lebov et al., 2017), to assess the risks posed by legacy mining contamination. By analyzing PTE concentrations in edible and forage crops, evaluating soil contamination and resilience, and linking these findings across health domains, we illustrate how abandoned mine tailings generate cascading impacts. Our study enhances the understanding of environmental and public health risks linked to legacy mining by integrating data on crops, soils, and contamination risk. It also strengthens the application of the One Health framework in managing contaminated agricultural landscapes.

## Material and methods

Study area. This study was conducted in the municipality of San Felipe de Jesús, which is situated along the Sonora River in northwestern Mexico (Fig. 1a). The town is positioned at 29.862312° N and 110. 238284° W, with an elevation of 853 m above sea level (masl). The climate in this region is classified as arid and semi-arid (BS0 and BS1) with average monthly temperatures ranging from 12.3 °C in January to 30.4 °C in July. However, during summer, maximum temperatures can reach 47 °C (Brito-Castillo et al., 2010). The annual precipitation is 481 mm, predominantly falling in July and August (SMN, 2023). The regional vegetation is thorn scrub, primarily dominated by legume trees and cacti (Martínez-Yrízar et al., 2010).

Mining operations in San Felipe de Jesús started approximately around 1900 with significant fluctuations in metal output; the primary metals extracted were lead and zinc, accompanied by smaller quantities of gold, silver, and copper. Built in 1973, a flotation plant was constructed with a capacity to process 100 tons per day (Roldan-Quintana 1979). Production of this plant involved processing ore from several mines in the region until it ceased in 1991 (Tietz, 2018). During the time frame of 1973 to 1991, the total output was roughly 104,000 tons, with typical compositions of 10.4% zinc, 2.6% lead, 0.3% copper, and 75.7 g of silver per ton. Ore processing resulted in a deposit of MTs adjacent to the flotation plant, between San Felipe and Aconchi (Espinoza-Madero, 2012).

The MT pile (Fig. 1b) measures approximately 200 × 300 m in size and spans an area of 16,300 m^2^ with heights ranging from 2 to 5 m (Del Rio-Salas et al. 2019; Espinoza-Madero, 2012); external surface is reddish (oxidized) while inner zones are grayish (mostly unoxidized). No vegetation has been observed colonizing the MTs since abandonment in the early 90 s without any remediation efforts. As a result, this pile has been a significant source of local pollution due to wind erosion during the dry season, which carries metal-enriched dust to the town of San Felipe and nearby agricultural soils (Fig. 1c). Furthermore, water erosion transports PTE during heavy summer rains, as the tailings are linked to the nearby Sonora River via a stream known as ¨El Lavadero¨ (Fig. 1c), which originates from Sierra Aconchi (Del Rio-Salas et al. 2019; Loredo-Portales et al., 2020). Agriculture and cattle raising are the most economically significant regional activities, surpassing the importance that mining had in the past (Del Rio-Salas et al. 2019). Agriculture is practiced along the flood plains of the Sonora River (Roldan-Quintana 1979) using irrigation water extracted from wells, a water spring (Huepac), or directly from the river. Previous studies have shown that most PTEs in surface and ground water in San Felipe are below the Mexican and international standards and are not likely a source of PTE into agricultural soils in the study area (Archundia et al., 2021, 2024). The major crops for human consumption include peanuts, maize, pepper and garlic; and alfalfa, barley and other grasses for livestock (SIAP, 2023). In this area, peanuts, maize and peppers are planted during July–August, whereas alfalfa and ryegrass in October and barley and garlic in November (INIFAP, 2017).

Collection and processing of crop and soil samples. Twenty-six fields (Online Resource S1), strategically distributed throughout the agricultural area, were carefully selected to encompass the most prevalent crops in San Felipe de Jesús (Fig. 1c). These fields varied in proximity to the MTs, with some situated near the MTs deposit to the south of San Felipe de Jesús, while others were close to the town, the Sonora River, and the northeast region of the town (Fig. 1c). Previous studies have shown that the concentration of PTEs decreases with distance from the mine tailings, with greater values within 1 km (Csavina et al., 2012). In our case, we covered a distance of up to 5 km, where depending on prevailing wind direction, background values are reached (Qi et al., 2022). To compare PTE concentrations in agricultural soils with regional background levels, we extracted data from the geochemistry layer available on the website of the Servicio Geológico Mexicano (https://www.sgm.gob.mx/GeoInfoMexGobMx/#), which provides information on regional soils. We selected soil data from sites located 10 to 20 km north and south of the mine tailings deposit and retrieved the reported concentrations. The mean background values from these sites are presented as reference values (RV) in Figure 3. In each field, five samples of the edible organ of the crop (leaves, fruits, or seeds) were collected, along with five top-soil samples from the root zone (0–20 cm in depth) of each collected plant. The collection of samples followed a random approach within each field, ensuring a minimum separation of 10–15 m between collection points. We focused on four crops intended for human consumption and four for cattle consumption. For human consumption, five fields of peanuts (*Arachis hypogaea* L.), two fields of Anaheim pepper (*Capsicum annuum* L.), two fields of maize (*Zea mays* L.), and one field of the non-domesticated pepper locally known as "chiltepin" (*Capsicum annuum* var. *aviculare* (Dierb.) D´Arcy and Eshbaugh) were selected. Mature fruits were collected from peanuts, Anaheim pepper, and chiltepin, while mature cobs were collected from maize plants. For forage crops, ten fields of the locally significant forage known as "zacatón" (*Sorghum halepense* L.), three fields of barley (*Hordeum vulgare* L.), two fields of alfalfa (*Medicago sativa* L.), and one field with a mixture of Rye grass (*Lolium perenne* L.) and barley were selected. In this case, above-ground organs (leaves and stems) were collected from each of the five selected plants. The unequal number of food and forage crop samples reflects the relative importance and availability of each crop in the agricultural area of San Felipe. Crop samples were placed in paper bags, and soil samples were sealed in plastic bags. Equal amounts of individual crop and soil samples from each field were combined to form a composite sample per field. A pilot test was conducted to determine whether there were differences in PTE concentrations between washed and unwashed forage samples of *Sorghum halepense* from two fields, and whether the composite sample concentration approximated the mean of five individual samples. The results (Online Resource S2 A) showed no significant differences in the concentrations of six key PTEs between washed and unwashed samples. In addition, the composite sample values were very close to the mean of the five individual samples and fell within one standard deviation. Based on these findings, we used unwashed forage samples and composite crop samples for each field. In the case of crops intended for human consumption, samples were washed with tap water to reflect typical household practices. In the laboratory, plant samples were sun-dried in closed paper bags, while soil samples were oven-dried. For soil samples, a pilot test was also conducted to evaluate variation within and between fields. In two fields (Sites 2 and 7), where five independent soil samples were collected, we partitioned the variance in PTE concentrations using a generalized mixed model. For all eight PTEs analyzed, the majority of the variance was associated with differences between fields rather than differences among samples within a field (Online Resource S2 B). While limited in scope, this finding suggests that compositing soil samples at the field level captures the dominant scale of variation in this system.

We acknowledge the limitation associated with compositing plant samples at the field level, which reduces the ability to assess intra-field variability in PTE concentrations. While our pilot test (Online resource S2 A) showed minimal variation in 6 key PTE between individual and composite samples of *Sorghum halepense*, and no significant differences between washed and unwashed forage samples, we recognize that this test was Limited in scope and may not fully represent the variability across all crop types and elements. The decision to composite samples was made to balance analytical costs, logistical feasibility, and to ensure consistency across the 26 sampled fields. Given the primary aim of this study -to assess broader spatial patterns of contamination and food safety risks across the agricultural area rather than within-field variability-, we prioritized spatial coverage and crop diversity over replication within fields.

Following the drying process, crop samples designated for human consumption underwent individual processing, focusing solely on the edible portions. The processing methodology varied for each crop type. For peanuts, the shells were discarded, and only the dried seeds from each sample were utilized. For peppers, the peduncle and calyx were removed, and only the dried fruits were employed. For maize cobs, only the grains were used. Each crop sample was ground in an electric mill (Cuisinart, model SG-104) and further pulverized in an agate mortar. For forage crops, solely the dried aerial parts were utilized. The dry tissue was cut into small pieces with scissors, ground in an electric mill (Cuisinart, model SG-104), and then pulverized in an agate mortar. Subsequently, both food crop and forage samples were securely stored in the laboratory until the commencement of the analysis. Regarding individual soil samples, once dried, they underwent sieving through a 1 mm sieve and were then stored in the laboratory awaiting analysis.

Analysis of soil properties and PTEs in soil and plant samples. In the investigation of soil properties, namely Electrical Conductivity (EC), pH, Organic Carbon (OC), and Cation Exchange Capacity (CEC), sieved soil samples from 26 fields were subjected to analysis. For EC and pH determination, a 3 g soil sample was combined with 30 mL distilled water in a 50 mL beaker, stirred for 18 h, and allowed to stand for an additional hour (Ponce de León et al., 2012). The pH and EC were quantified using a VWR Symphony B30PCI pH and conductivity meter. To assess OC, carbonates were first removed using 5 N HCl. Subsequently, samples were subjected to complete combustion in an elemental analyzer (CHNS/O Thermo Scientific Flash 2000), utilizing aspartic acid as a calibration standard. CEC was evaluated through extraction with a 0.5 M ammonium chloride solution. Ca and Mg content were determined using a Perkin Elmer Atomic Absorption Spectrophotometer PinAAcle 900H, while K and Na were determined using a Sherwood 360 flame spectrometer.

Total concentration of PTEs were measured in soil samples and in the edible parts of plant samples using Inductively Coupled Plasma Mass Spectrometry (ICP-MS) at a certified laboratory (Australian Laboratory Services, ALS, Vancouver, Canada). Soil samples (0.5 g) underwent digestion with 75% aqua regia (3:1 HCl: HNO3) in a graphite heating block. The final solution was then analyzed by ICP-MS using the ME-MS41L method at ALS. To ascertain accuracy and precision (± 10%), OREAS 905 and MRGeo08 standards were employed as references. For plant samples, fine powder (1 g) was subject to cold digestion with HNO3 for 8 h, followed by heating for 15 min at 85 °C and an additional 2 h at 115 °C. After cooling, the samples were brought to volume with HCl, and the resulting solution was analyzed by ICP-MS at ALS, utilizing the ME-VEG41 method. Accuracy and precision (± 10%) were established using NIST-1515 and NIST-1575a standards. The Quality Assurance/Quality Control (QA/QC) procedure at ALS was based on blanks, reference standards and duplicate samples; detection Limits were 0.01, 0.001, 0.01, 0.1, 0.005, 0.1 mg kg⁻^1^ for As, Cd, Cu, Mn, Pb, and Zn, respectively and 0.01, 0.001, 0.01, 0.001 and 0.01% for Ca, Fe, K, P and S, respectively.

Estimation of soil resilience index. Song et al. (2022), proposed that soil resilience to pollution can be reflected by a set of key physical and chemical indicators. We followed their approach to estimate soil resilience using five specific soil indicators: OC, CEC, pH, TFe_2_O_3_ (total iron oxides) and CaO (calcium oxides). OC, CEC and pH were estimated as previously described. For TFe_2_O_3_ and CaO, we used oxide conversion factors to convert Fe (%) and Ca (%) to TFe2O3 (1.4297) and CaO (1.3992), through stoichiometric conversion (https://www.jcu.edu.au/advanced-analytical-centre/resources/element-to-stoichiometric-oxide-conversion-factors). For every soil sample, each indicator was scored as poor (1), medium (2), good (3), or excellent (4) according to threshold values reported by Song et al. (2022): OC (< 0.6%, 0.6–1.2%, 1.2–2.0%, > 2.0%); pH (< 5.5, 5.5–6.5, 6.5–7.5, > 7.5); CEC (< 10, 10–20, 20–30, > 30 cmol/kg); Fe₂O₃ (< 2%, 2–5%, 5–8%, > 8%); and CaO (< 0.5%, 0.5–1.0%, 1.0–2.0%, > 2.0%). Scores for each field were summed to obtain a composite resilience index, with low totals indicating weak resilience (limited ability of soil properties to buffer heavy metal stress) and high totals indicating strong resilience (greater capacity to sustain agricultural production and protect groundwater quality). If PTE concentration in crops were above safe values for human or animal consumption, we explored whether they were associated with poor soil resilience or not.

Statistical analysis. Normal distribution of the total concentration of PTE in both plants and soils was assessed using the Shapiro-Wilks test in R (R Core Team, 2023). Skewed variables were transformed to common logarithms (log10) to stabilize their variances for subsequent analysis. We analyzed the Pearson correlation coefficients among soil properties (CEC, EC, pH, organic C) location factors (distance to mine tailings deposit and Sonora River) and the concentration of PTEs in soils (Pb, As, Zn, Mn, Cd, etc.), and edible parts of plants (Pb, As, Zn, Mn, Cd, etc.). Due to differences in variable scales and the inclusion of both transformed and untrasformed data, a Principal Component Analysis (PCA) was conducted on the correlation matrix to explore patterns and associations among PTE concentrations in soils and plants. For the pilot tests, generalized Linear models were used in JMP ver. 18. The relationship between the concentration of some PTE in soil samples and the distance (in km) from the mine tailings deposit was evaluated using the global curve fitting function of Sigma Plot ver. 14 (Systate Software, 2018).

## Results

**Fig. 2:**
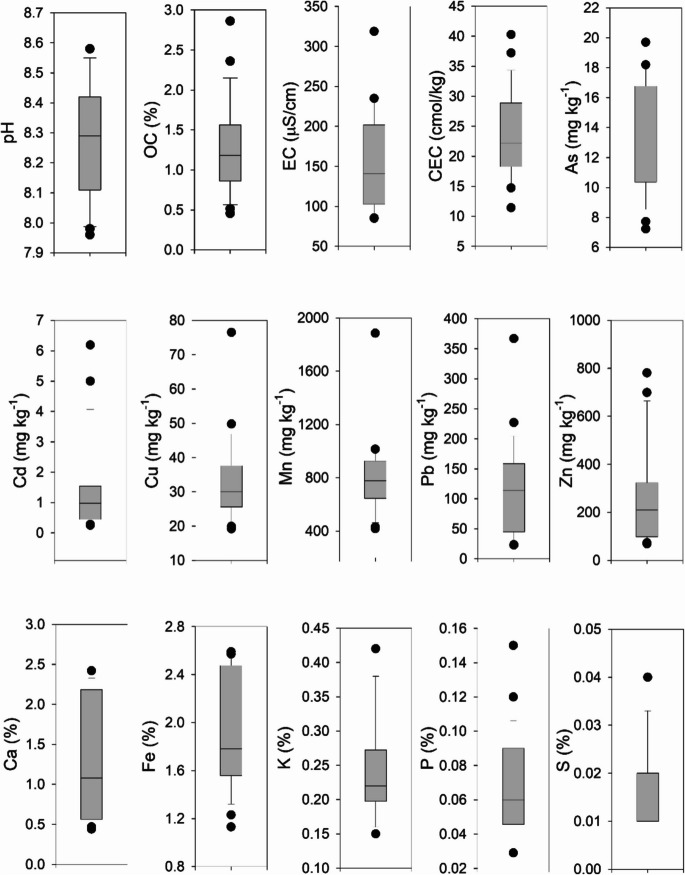
Box plots showing some of the soil properties measured in the study area. Soil properties include pH, Organic carbon (OC), Electrical Conductivity (EC), Cationic Exchange Capacity (CEC), and the concentration of As, Cd, Cu, Mn, Pb, Zn, Ca, Fe, K, P and S

Soil properties. The agricultural soils examined in San Felipe showed a slightly alkaline nature, with pH values ranging from 7.96 to 8.58. Salinity levels, as indicated by electrical conductivity (EC), were relatively low, spanning from 85.07 to 318.66 µS/cm. OC content varied from 0.45% to 2.86%, reflecting a range from low to moderate. CEC exhibited variation from medium to high, with values ranging between 11.41 and 40.28 cmol/kg across different sites (see Fig. 2 and Online Resource S3).

In terms of potentially toxic elements, arsenic (As) total concentrations ranged from 7.23 to 19.70 mg kg⁻^1^, while cadmium (Cd) exhibited variation from 0.013 to 6.19 mg kg⁻^1^. Copper (Cu) levels ranged from 19.2 to 49.8 mg kg⁻^1^, manganese (Mn) showed variability from 418 to 1885 mg kg⁻^1^, and lead (Pb) concentrations ranged from 22.70 to 367 mg kg⁻^1^. Zinc (Zn) concentrations varied from 68.4 to 781 mg kg⁻^1^ (see Fig. 2).

Other elements such as calcium (Ca) ranged from 0.44% to 2.42%, iron (Fe) varied from 1.13% to 2.59%, potassium (K) ranged from 0.15% to 0.42%, phosphorus (P) exhibited variability from 0.029% to 0.15%, while sulfur (S) showed concentrations ranging from 0.01% to 0.04% across the analyzed fields (see Fig. 2).

Relationships between PTE and soil properties.—In general, soil properties exhibit a low correlation with the concentration of PTE in soils. Noteworthy instances include the negative correlation between soil pH and the concentration of K, S, Cu, Cd, and Zn in soils (Online Resource S4), while electrical conductivity (EC) only has a positive correlation with S in soils. Organic carbon (OC) shows a strong relationship with soil K and S, while cation exchange capacity (CEC) is positively correlated with Ca, P, As, Fe, K and S in soils (Online Resource S4).

Strong correlations were also detected among different PTE in soils (Online Resource S4). PCA of the concentration of PTE in soils (Online Resource S5) show that Ca, Fe, and P share a common pattern of variation; K and S form another group, and a third one is integrated by Pb, Zn, Cd. In these principal components Lies more than the 90% of variance (Online Resource S6).

**Fig. 3:**
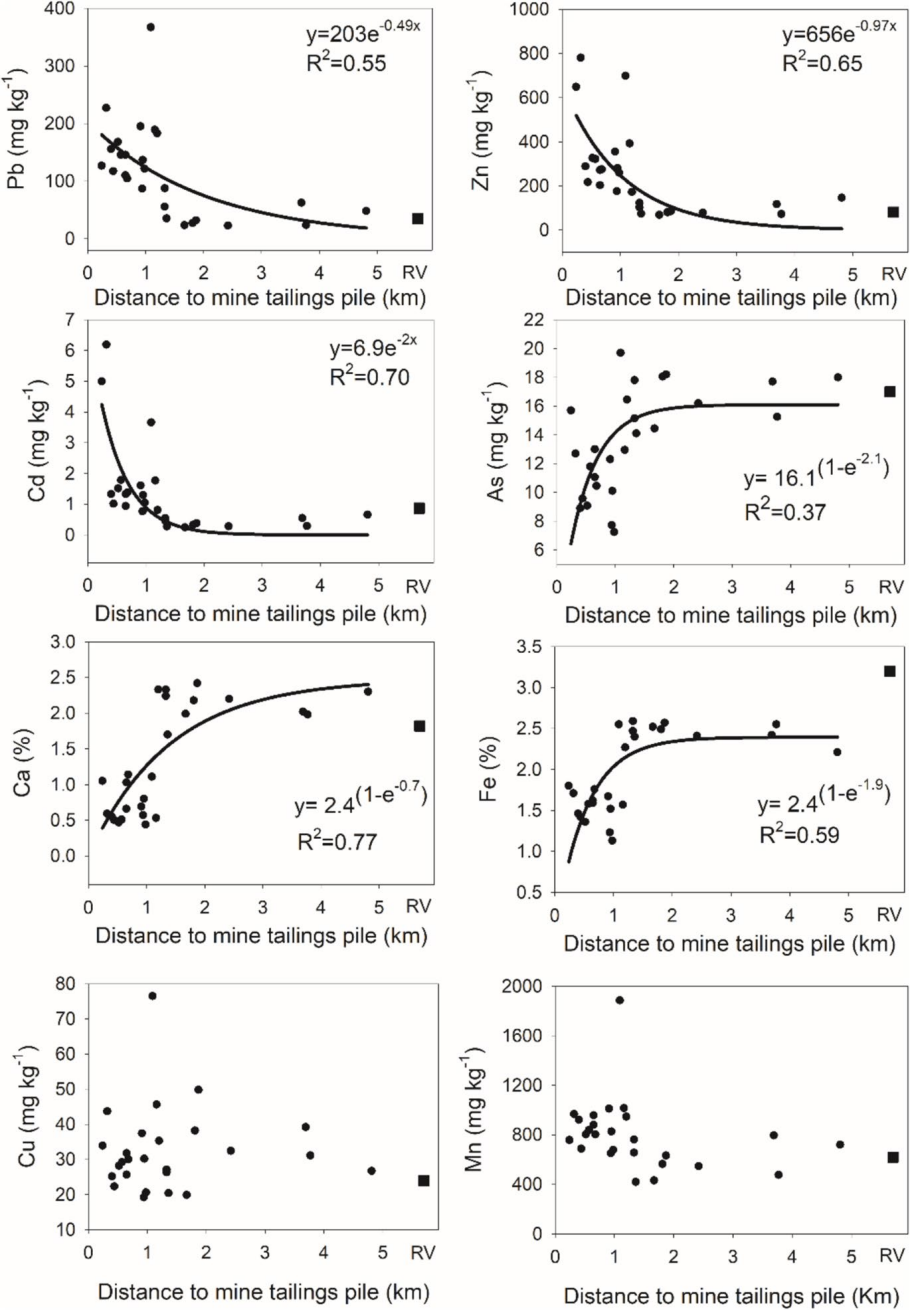
Spatial patterns in the concentration of PTEs as a function of distance from the abandoned mine tailings. For Pb, Zn and Cd, a negative exponential decline was recorded. Each plot shows the best fit to an exponential decay equation. For As, Ca and Fe, a positive exponential increase to a maximum was recorded. Each plot shows the best fit to an exponential rise to a maximum equation. For, Cu and Mn, no significant spatial pattern was recorded. Square symbols indicate background levels obtained from geochemistry layer of the Servicio Geológico Mexicano (https://www.sgm.gob.mx/GeoInfoMexGobMx/#) whereas filled circles indicate observed values

Spatial patterns in PTE concentration. The concentration of certain elements exhibited distinctive spatial patterns with distance from the mine tailings (see Fig. 3). Notably, elements such as Pb, Zn, and Cd, which are prevalent in relatively high concentrations within the mine tailings (Del Río-Salas et al., 2019), displayed an exponential decline as distance from the mine tailings increased until reaching regional background values (Fig. 3). Conversely, other elements exhibited an enrichment pattern with increasing distance from the mine tailings. This trend was observed in elements such as As, Ca, and Fe, which showed an exponential increase until reaching a peak at distances of 2 to 4 km from the mine tailings. In contrast, elements like Cu and Mn, despite being in significant concentrations in the tailing pile (Del Rio-Salas et al. 2019), displayed no clear pattern with distance (Fig. 3).

**Fig. 4:**
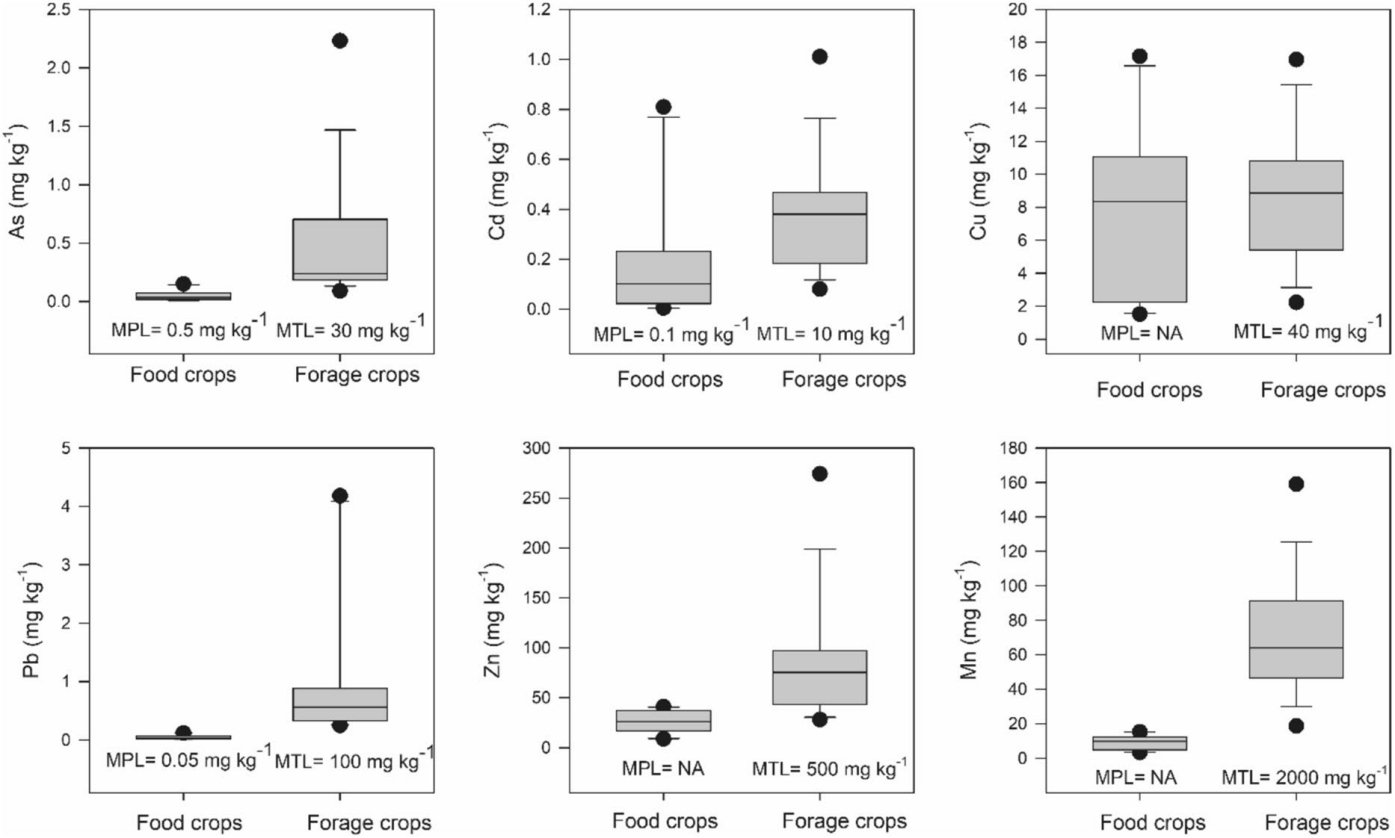
Box plots showing variation in the concentration of potentially toxic elements in leaves and stems of forage crops and fruits and seeds of food crops. In each plot, the maximum permissible level (MPL) of food crops for human consumption (FAO/WHO, 2001) and the maximum tolerable levels (MTL) of forage crops for cattle consumption (National Research Council, 2005) is shown. NA: Non-available

**Fig. 5:**
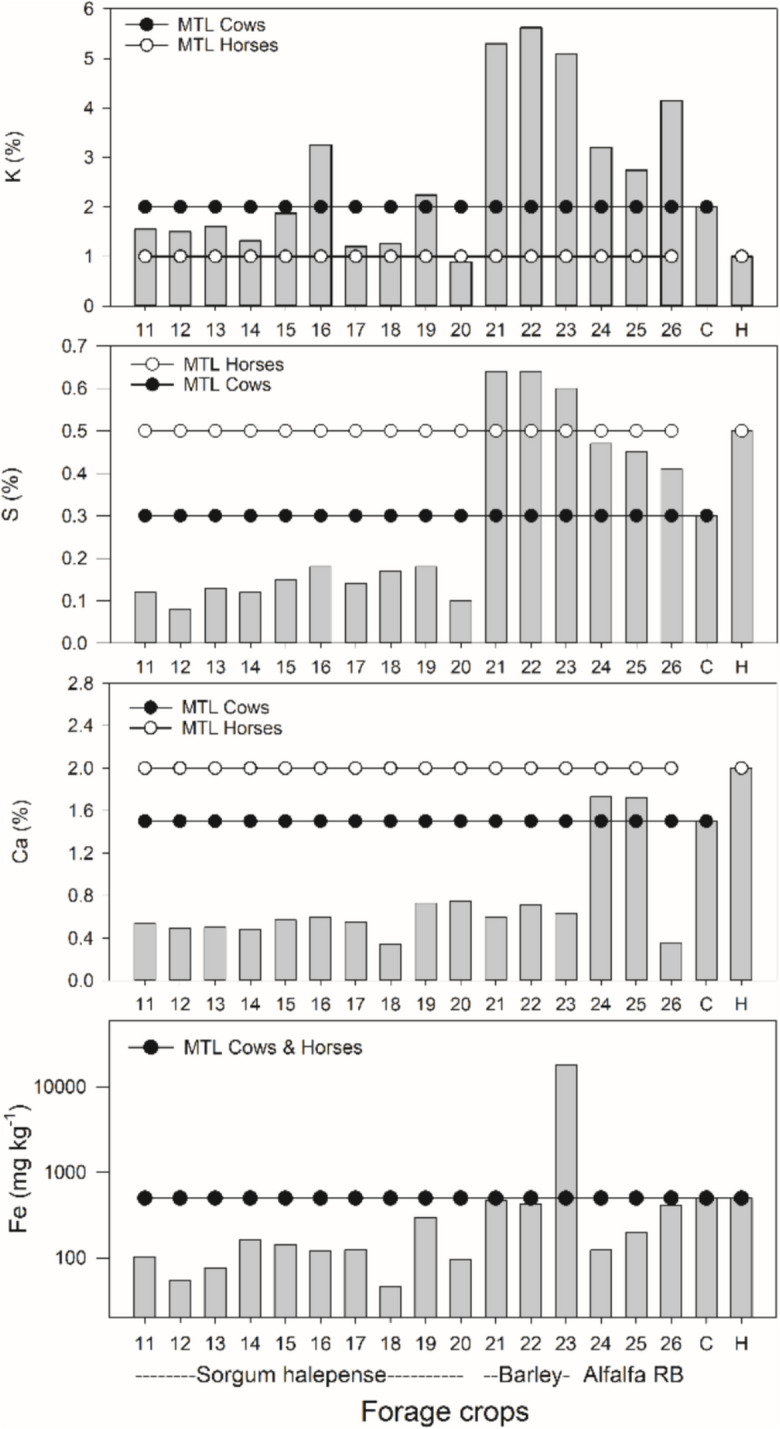
Concentration of K (%), S (%), Ca (%) and Fe (mg kg.^−1^) among forage crops from San Felipe de Jesús. C and H indicate the MTL for cows and horses (National Research Council, 2005)

PTE accumulation in crops. Generally, forage crops exhibited a higher concentration of PTEs in the palatable tissues (leaves and stems) compared to the palatable organs (fruits, seeds) of crops intended for human consumption (see Fig. 4). As in soils, several elements were correlated within plants (Online Resource S4). PCA for the concentration of these elements showed that Fe, Zn and As shared a similar pattern and Ca and Cd formed another group (Online Resource S7). In these two leading components Lies close to 69% of the variance (Online Resource S8). For forage crops, the majority of elements remained below the maximum tolerable levels (MTL) for cows and horses (Fig. 4, National Research Council 2005). However, concentration of K, S, Ca and Fe in some or all barley and alfalfa samples exceeded the MTL for either one or both animal species (Fig. 5). For K, all the analyzed barley and alfalfa samples exceeded the MTL for horses and cows, whereas for S only barley samples exceeded MTL for cows (Fig. 5).

**Fig. 6:**
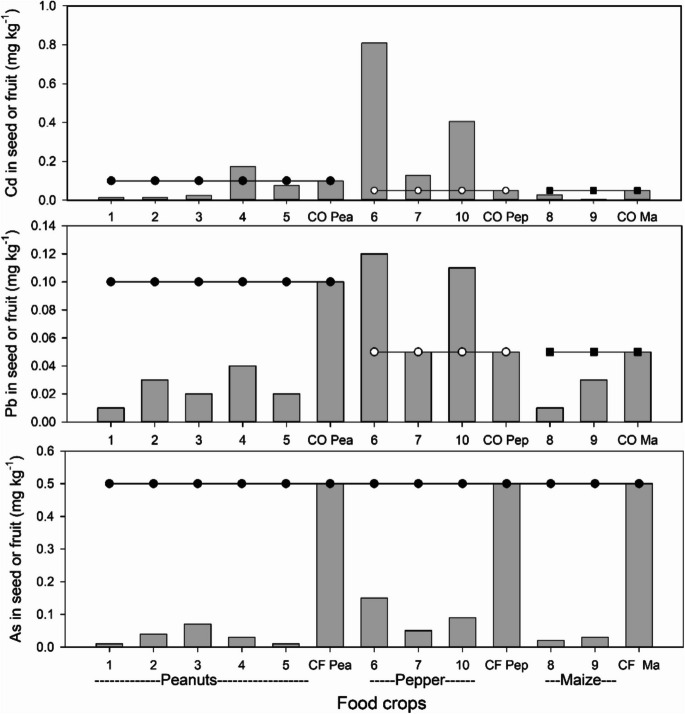
Concentration of Cd (mg kg^−1^), Pb (mg kg^−1^) and As (mg kg^.1^) among food crops for human consumption in San Felipe de Jesús. CO Pea, CO Pep and Co Ma show the MPL for peanuts, peppers and maize according to the Codex Alimentarius (FAO/WHO, 2001). CF Pea, CF Pep and CF Ma show the MPL for peanuts, peppers and maize according to the limits of contaminants in Food in the Chinese Legislation (NHFPCPRC & CFDA 2017). Sample 10 corresponds to chiltepin

Among the food crops, a single sample of peanuts (4 in Fig. 1), the two pepper samples (6 and 7 in Fig. 1), and the chiltepin sample (10 in Fig. 1) exceeded the Codex Alimentarius limits (FAO/WHO, 2001) for Cadmium (Cd) in peanuts (0.1 mg kg⁻^1^) and peppers (0.05 mg kg⁻^1^, Fig. 6). Conversely, both maize samples had Cd concentrations below the Codex Alimentarius limit (0.05 mg kg⁻^1^, FAO/WHO, 2001). For Lead (Pb), all peanut and maize samples remained below the limit (Fig. 6). However, one pepper sample (6 in Fig. 1) and the chiltepin sample (10 in Fig. 1) exceeded the Codex limits (0.05 mg kg⁻^1^, Fig. 6). Lastly, for Arsenic (As), all analyzed food crops fell below the maximum limit established (0.5 mg kg⁻^1^) by the Chinese legislation (Fig. 6, NHFPCPRC & CFDA 2017). Bioconcentration factors (BCF = [crop]/[soil]) for Cd and Pb, the only PTEs that exceeded Codex Alimentarius limits in food crops were very low (< 0.01), indicating that these crops are not efficient accumulators.

Soil resilience. – Based on the sum of scores from five of the soil indicators proposed by Song et al. (2022) to evaluate resilience, 65.4% and 34.6% of the samples from San Felipe were scored with moderate and poor soil resilience, respectively (Online Resource S9). For food crops, two samples where PTE concentration were above safe standards (6 and 10) were from soils with moderate resilience whereas one (4) was from a soil with poor resilience (Online Resource S9). For forage crops, three samples where PTE concentration were above safe standards were from soils with moderate resilience and another three samples from soils with poor resilience (Online Resource S9).

Correlation of Cd and Pb in soil and crops. In general, few significant correlations were recorded between PTE in soil and crop samples (Online Resource S4). Contrasting correlations were observed for the two elements that were above the Codex Alimentarius limit in food crops. A positive and statistically significant correlation was observed between the concentration of Cd in leaves (forage crops) and fruits/seeds (food crops) and the corresponding concentration of Cd in soil (Online Resource S10). In contrast, no significant correlation was detected for Pb between its concentrations in leaves (forage crops) and fruits/seeds (food crops) and the concentrations in soil (Online Resource S10).

## Discussion

Agricultural soils of San Felipe exhibit moderate (65.4%) to poor (34.6) resilience, characterized by low levels of organic carbon (OC), medium to high CEC and low electrical conductivity (EC). While EC is commonly used as an indicator of the ionic strength of the soil solution, it does not directly measure metal ions. In this case the observed low EC values combined with higher CEC suggest that, under acidic conditions, there may be an increased potential for the mobilization and lixiviation of PTE into the soil solution. This could result in the release of PTE that are more readily available for plant uptake. Supporting this, the negative correlation observed between soil pH and the concentrations of Cu, Cd, and Zn indicates that these elements are more likely to be released under acidic conditions, which can increase their bioavailability in the rhizosphere. Notably, certain samples surpass the threshold total concentrations in soils known to induce phytotoxic effects for Zn and Pb (160 and 20 mg kg^−1^, respectively) (Bucher & Schenk, 2000; Menzies et al., 2007; Mertens & Smolders, 2013).

The observed correlation of As, with cation exchange capacity (CEC), may suggest an indirect association with iron oxyhydroxides, which are known to retain cations like arsenate under slightly alkaline pH conditions. Although this mechanism has been proposed in previous studies from the region (Loredo-Portales et al., 2020), it could not be directly evaluated here, as this study only measured total metal concentrations in soils. Similarly, the strong correlation among Pb, Zn, and Cd in soils may reflect their shared mobility potential, as these PTE commonly occur as divalent cations in the soil solution. Although this study did not assess PTE speciation, previous research in the region has shown that these elements can be present in labile fractions (including soluble, exchangeable, and carbonate-bound) in agricultural soils, accounting for up to 75% of their total concentration (Del Rio Salas et al. 2019); Loredo-Portales et al., 2020). Future studies should incorporate speciation analyses and consider additional factors such as redox potential, which can significantly influence the behavior and availability of PTE in soils (Young, 2013).

Previous studies have reported a gradual decline in PTEs with increasing distance from mine tailings or smelters, consistent with eolian transport as the primary dispersal mechanism (Kim et al., 2014; Li et al., 2017). At our study site, Pb, Zn, and Cd exhibited an exponential decline with distance from the MTs (Fig. 3), aligning with their high concentrations in the tailing pile (Del Rio-Salas et al. 2019), and supporting wind transport as a likely vector. Meteorological data from the last decade (2013–2023) in nearby towns (Baviácora and Banámichi, see Fig. 1) and a recently established station in San Felipe de Jesús reveal a prevailing N-NE wind direction at speeds of 7–21 km/h (CESAVE-SIAFESON, 2023), consistent with the observed spatial patterns.

In contrast, As, Ca, and Fe, exhibited an enrichment pattern with distance from the mine tailings, with concentrations rising as distance from the tailings increased (Fig. 3). For instance, Ca concentrations were lowest near the tailings and increased with distance (as also reported by Gonzalez-Mendez et al. 2022), showing negative correlations with Cd (r = −0.50, P = 0.007) Pb (r = −0.59, P = 0.001), and Zn (r = −0.60, P = 0.0008). This pattern suggests a potential role of calcium -likely as carbonates- in metal retention, since carbonate-rich soils can immobilize metals through precipitation reactions (e.g. forming metal carbonates), but also helps to maintain the alkaline pH that affect PTE solubility and enhance the soil’s buffering capacity (Madrid and Diaz-Barrientos 1992). As a result, near the tailings where potentially calcium carbonate content is lower, metals could remain more mobile, while farther from the tailings, the increase in Ca, -likely as carbonate- may reduce metal availability. Therefore, calcium carbonate could play a key role in the resilience of soils, helping them to recover from contamination and limiting the impact of toxic elements on plant growth and ecosystem health (Wang et al., 2015). Furthermore, the positive correlation between soil Ca and Fe (r = 0.90, P < 0.0001) likely contributes to the similar spatial distribution observed for both elements. Although arsenic is present in mine tailings at significant concentrations, it is mainly associated with residual fractions, iron and manganese oxides, and coarse sand and silt particles, which may limit its dispersion and vailability in agricultural soils (Loredo-Portales et al., 2020). Despite the high concentrations of As in the tailings and nearby soils, elevated levels are also found along both sides of the Sonora River, likely due to upstream mine spills (Gonzalez-Mendez et al. 2022). Thus, the spatial distribution of As probably reflects the influence of the Sonora River. Finally, Cu and Mn concentrations showed no discernible patterns with increasing distances from the tailings pile (Fig. 3).

Among the forage crops analyzed, most samples were below the Maximum Tolerable Levels (MTLs) for cows and horses. However, barley and alfalfa samples exceeded the MTLs for K for both animal species whereas for S both forage species surpassed MTL for cows, while one barley and two alfalfa samples surpassed the MTLs for Fe and Ca (Fig. 5). Although elevated levels of these elements have been reported in forage species growing near mines, their accumulation in plant tissues is often unrelated to direct mine pollution (Lottermoser, 2011). In this study, the spatial distribution of samples exceeding MTLs for K, S, Ca, and Fe does not indicate a clear association with proximity to the mine tailings pile. However, S may play a role in plant metal tolerance (Cao et al., 2023; Ren et al., 2017), and in this study, S was positively correlated with concentrations of Pb (r = 0.74, P < 0.0001), Zn (r = 0.62, P = 0.0006), Cu (r = 0.72, P < 0.0001), and As (r = 0.72, P < 0.0001). This suggests that elevated S levels in some forage samples may be indirectly linked to metal pollution in San Felipe's agricultural soils. Excessive S in forage can impair mineral absorption, hinder growth, and induce toxicity in ruminants (Drewnoski et al., 2014; Kandylis, 1983). Similarly, high concentrations of Fe and K may interfere with mineral metabolism and pose direct health risks to livestock (Masters et al., 2018; Wysocka et al., 2020). The presence of forage crops exceeding MTLs for these elements highlights the need for management strategies to reduce potential risks to animal health. As a preventive measure, it is advisable to analyze the excreta and hair of domestic animals in San Felipe (Madejón et al., 2009) to assess possible health effects from consuming contaminated forage.

For food crops, several fields, particularly those cultivating peanuts and peppers, exhibited Cd levels above the Codex Alimentarius limit (FAO/WHO, 2001); in addition, peppers also showed Pb levels above the permissible limit (Fig. 6). These fields are situated within 1 km from the tailings pile, suggesting an association with the high concentrations of Cd and Pb in the soil near the pile (Fig. 3). Notably, the accumulation of Cd in peanut seeds exceeding permissible limits has been observed in polluted soils in China, where a positive and significant correlation was observed between soil Cd levels and seed concentration, as in this study (Yang et al., 2022). Similarly, instances of Pb and Cd accumulation in pepper fruits surpassing maximum limits have been reported in contaminated agricultural soils in Brazil and Cuba (Rodriguez-Alfaro et al. 2022; Santos Silva et al. 2019). The ingestion of food crops with elevated levels of Pb and Cd has been linked to carcinogenic effects and various human health issues, including neurological and cardiovascular diseases, kidney dysfunction, hypertension, and other complications affecting the liver, lungs, nervous system, and immune system (Munzel et al. 2023; Rai et al., 2019). Therefore, to gauge the transfer of PTEs from food crops in the agricultural soils of San Felipe to humans, it is imperative to employ diverse health risk assessment indices (Rai et al., 2019). Such assessments are crucial for implementing appropriate measures if health risks are high.

Cases where food and forage crops showed PTE values above safe standards were not limited to soils with poor resilience values. In fact, more than half of the exceedance occurred in soils classified as having moderate resilience, indicating that other factors may influence the transfer of PTE from soil to the edible part of crops. While soil resilience -defined here by indicators like pH, EC, organic carbon, and CEC- can reflect a soil’s buffering capacity (Song et al., 2022), it may not fully account for all mechanisms governing PTE retention. Recent evidence points to the critical role of soil carbonates in regulating PTE bioavailability. For example, Wang et al. (2015) found that carbonate depletion, even without substantial changes in pH, resulted in to 2–3 times higher Cd and Ni concentrations in wheat grains. This highlights the carbonate content, independent of pH, can be a decisive factor in metal uptake. Thus, although conventional resilience indicators offer valuable insights, the presence or absence of carbonate minerals may play a key -but often overlooked- role. Future assessments of soil resilience and contamination risk in arid, mining-impacted agricultural areas should consider carbonate content as a key variable influencing both ecological vulnerability and food safety.

In addition, PTE accumulation in crops depends not only on soil properties but also on plant-specific bioaccumulation capacities (Liu et al., 2023). For instance, peanuts have a strong ability to bioaccumulate Cd and Pb in seeds (Zhang et al., 2024). Thus, while a soil buffering capacity contributes to its resilience against PTE pollution (Song et al., 2022), other processes regulating the transfer of PTEs in the soil-crop system (Liu et al., 2023) may be also important.

With food safety being a paramount concern for human health (Gizaw, 2019), effective management of soil pollution in the agricultural soils of San Felipe requires the implementation of at least two strategies: source reduction and remediation of soils in close proximity to the tailings pile. Given that the abandoned tailings pile stands out as the primary local source of PTEs in the agricultural soils of San Felipe, a phytoremediation program is imperative to curtail the transfer of contaminants to the surrounding soils. A recent study that evaluated various local native plant species and phytostabilization strategies, concluded that a soil capping approach is the most effective (Borbon-Palomares et al. 2023). Therefore, the initial step should involve the implementation of a phytostabilization program in the abandoned tailings, utilizing a soil cap. The agricultural soils in close proximity to the abandoned mine tailings exhibit PTE levels above national and international standards and are phytoaccessible (Del Rio-Salas et al. 2019; Loredo-Portales et al., 2020). Hence, even with a reduction in the source of PTEs, adopting remediation strategies aimed at diminishing the transfer of pollutants from soil to crop becomes imperative, such as the use of amendments (Abkar et al., 2024).

Our findings also align with global efforts to reduce toxic element exposure through food. For example, the US Food and Drug Administration (FDA) launched the Closer to Zero initiative in 2021, aiming to reduce the concentration of Pb, As, Cd and Hg in foods consumed by infants and young children (US FDA, 2021). Although this initiative is US-based, its principles are relevant worldwide. The detection of Cd and Pb concentrations exceeding international limits in locally grown peanuts, chiltepin and peppers in our study highlights a broader food safety concern with cross-border implications. These results underscore the importance of coordinated international monitoring and risk reduction strategies, especially in vulnerable rural and agricultural regions where environmental legacy pollution persists.

As previously mentioned, soil health is fundamental to produce safe and nutritious foods (FAO & UNEP 2021). Soil degradation through the transfer of PTEs -as seen in San Felipe- is one of the most ecological hostile legacies of mining (Cross et al., 2017). Combined with the low to moderate resilience of the local soils, this degradation leaves a lasting toxic footprint on ecosystems and poses serious risks to human well-being (Mendez & Maier, 2008; McHale et al. 2025). The level of pollution that has been documented in this as well as in previous studies (Loredo-Portales et al., 2020, Gonzalez-Mendez et al. 2022) show the potential human health risks stemming from the consumption of metal-contaminated food from San Felipe.

Our study underscores how abandoned mine tailings represent not only a localized source of contamination but also a broader One Health challenge. For human health, exceedances of Codex Alimentarius limits in peppers, chiltepin, and peanuts indicate direct food safety risks for local and regional consumers. For animal health, elevated levels of macroelements in forage crops may lead to nutritional imbalances in livestock, with potential impacts on local subsistence. Equally important are the environmental implications: reduced soil resilience, especially in organic carbon and cation exchange capacity, leaves these agricultural soils more vulnerable to degradation, while wind-driven dispersal of Pb, Zn, and Cd extends contamination beyond the tailing’s footprint. These combined pressures jeopardize ecosystem services and agroecosystem sustainability. By integrating these dimensions, our work illustrates how disturbances initiated by mining legacies cascade across all three domains of One Health. Effective responses should therefore include both exposure reduction and remediation of agricultural soils, ensuring that human, animal, and environmental health are addressed simultaneously.

Instances such as the abandoned mine tailings in San Felipe emphasize the significance of the One Health approach to food safety, recognizing the interconnectedness of human and environmental health (FAO, UNEP, WHO & WOAH 2022) through the role of soil and crop health in food safety. Polluted agricultural soils can precipitate systemic food security problems in the food supply chain, subsequently impacting human health (Vargas-Rojas et al., 2016). The adoption of sustainable soil management practices, including the implementation of a comprehensive remediation program in San Felipe, stands as a crucial measure to alleviate the impact of soil pollution on human health. Considering the socioecological problems caused by previous mine spills (Ibarra-Barreras and Moreno-Vazquez 2017) and the passive dispersal of PTE from mine waste such as in San Felipe, assessing the human health risk posed by crops grown in the Sonora River basin is imperative.

## Conclusions

This study shows that the abandoned mine tailings near San Felipe de Jesús serve as a persistent source of potentially toxic elements (PTEs), particularly Pb, Zn, and Cd, which disperse through wind erosion and accumulate in agricultural soils and crops. Although most forage crops were below the maximum tolerable levels, barley and alfalfa samples showed elevated levels of K, S, Ca, and Fe. In food crops, peanuts, peppers, and chiltepin exceeded international limits for Cd and Pb, indicating potential health risks.

Despite elevated PTE concentrations and moderate to poor soil resilience, the accumulation of PTE in most crops remained below the maximum tolerable levels. This suggests not only a complex interaction between soil properties and crop-specific uptake, but also that certain soil mechanisms -possibly linked to carbonate content or metal binding with oxides- may still be limiting the transfer of PTEs to plants. These unmeasured or underexplored factors may contribute to a form of resilience that buffers PTE uptake. Identifying and understanding these mechanisms will be essential for improving soil health and refining resilience assessments in contaminated arid soils.

To mitigate these risks, a dual strategy is needed: phytostabilization of the tailings to prevent further dispersion and amendment-based soil remediation to reduce PTE uptake by crops. These findings highlight the importance of integrating the One Health framework -recognizing the interconnectedness of environmental, animal and human health- to address soil contamination, food safety, and public health in mining-impacted agricultural regions. The effective management of mining legacies and the remediation of polluted agricultural soils are crucial steps in ensuring sustainable food production and public health.

## Supplementary Information

Below is the link to the electronic supplementary material.

Supplementary file1 (PDF 690 KB)

## Data Availability

No datasets were generated or analysed during the current study.

## Abbreviations

MTs: Mine tailings
PTE: Potentially toxic elements
PCA: Principal component analysis
MTL: Maximum tolerable levels
MPL: Maximum permissible levels
