# Supplementary material for: Evaluating potentially toxic element accumulation in crops near abandoned mine tailings in northwestern Mexico: a One Health perspective
Source: Environ Monit Assess. 2025 Sep 20;197(10):1126. doi: 10.1007/s10661-025-14587-1 (PMC12449420; doi:10.1007/s10661-025-14587-1)

Online Supplementary Material

Evaluating potentially toxic element accumulation in crops near abandoned mine tailings in northwestern Mexico: a One Health perspective

Environmental Monitoring and Assessment

Martha Camacho-Alcantar[1], Blanca González-Méndez[2,3], René Loredo-Portales[4], José Raúl Romo-León[5] and Francisco Molina-Freaner[6]*

[1]Posgrado en Biociencias, Universidad de Sonora, Hermosillo, Sonora, México

[2]CONAHCYT, Insurgentes Sur 1582, Ciudad de México, 03940, México

[3, 4]Estacion Regional del Noroeste, Instituto de Geología, Universidad Nacional Autónoma de México, Hermosillo, Sonora, México

[5]Departamento de Investigaciones Científicas y Tecnológicas, Universidad de Sonora, Hermosillo, Sonora, México

[6]Instituto de Ecología, Universidad Nacional Autónoma de México, Hermosillo, Sonora, México

*freaner@unam.mx

Online Resource S1. Location of the sampled fields in the agricultural area of San Felipe de Jesús, Sonora, México.

**Code**	**Crop**	**Latitude**	**Longitude**	**Altitude**
Crop samples for human consumption				
1	Peanut	29°51´23.4´´N	110°14´07.6´´ W	595 masl
2	Peanut	29°50´48.2´´N	110°14´36.3´´W	602 masl
3	Peanut	29°51´24.9´´N	110°13´55´´W	599 masl
4	Peanut	29°51´07.6´´N	110°14´33.5´´W	601 masl
5	Peanut	29°50´36.3´´N	110°14´40.1´´W	594 masl
6	Pepper	29°50´49.8´´N	110°14´44.7´´W	600 masl
7	Pepper	29°51´27.7´´N	110°14´14´´W	597 masl
8	Maize	29°50´46.5´´N	110°14´15.1´´W	598 masl
9	Maize	29°51´27.1´´N	110°14´12.5´´W	595 masl
10	Chiltepin	29°50´48.7´´N	110°14´41.5´´W	598 masl
Forage samples for cattle consumption				
11	*Sorghum halepense*	29°50´44.1´´N	110°14´29´´W	598 masl
12	*Sorghum halepense*	29°51´02.2´´N	110°14´34´´W	601 masl
13	*Sorghum halepense*	29°51´27.7´´N	110°14´18.6´´W	596 masl
14	*Sorghum halepense*	29°51´23.3´´N	110°13´48.1´´W	598 masl
15	*Sorghum halepense*	29°50´47.8´´N	110°14´16.4´´W	594 masl
16	*Sorghum halepense*	29°52´43´´N	110°13´44.7´´W	603 masl
17	*Sorghum halepense*	29°52´41.7´´N	110°13´37.2´´W	603 masl
18	*Sorghum halepense*	29°51´08.1´´N	110°14´10.5´´W	595 masl
19	*Sorghum halepense*	29°51´51.5´´N	110°13´43.9´´W	601 masl
20	*Sorghum halepense*	29°53´06.4´´N	110°13´07.8´´W	614 masl
21	Barley	29°51´14.3´´N	110°14´35.9´´W	599 masl
22	Barley	29°51´02.7´´N	110°14´12.9´´W	601 masl
23	Barley	29°50´36.3´´N	110°14´39.9´´	594 masl
24	Alfalfa	29°50´49.7´´N	110°14´15´´W	595 masl
25	Alfalfa	29°50´56.8´´N	110°14´05.4´´W	594 masl
26	Rye grass-barley	29°51´23.3´´N	110°13´45.5´´W	601 masl

Altitude in meters above sea level, masl.

Online Resource S2.

A) Results of the pilot test comparing the concentration (ppm) of 6 PTE between five plants of zacaton (*Sorghum halepense*) that were washed (**W**) with water (n=5) and unwashed (**UW**) in two sites in the agricultural area of San Felipe de Jesus in Sonora, Mexico. Site 2 was closer (0.4 km) whereas site 7 was farther away (3.77 km) from the mine tailings pile. Generalized linear models (normal distribution) were used to contrast the concentration of PTE in washed (**W**) and unwashed (**UW**) samples. For each element, we show the $\chi^2$and P value that indicate that there were no statistically significant differences between washed and unwashed samples. The last column shows the concentration of each element in composite samples formed by equal parts of five individual plants from each agricultural field. The values recorded in the composite samples indicate that they were within the mean ± SD of the five unwashed samples.

**Element**	**Site**	**Mean ± SD (W)**	**Mean ± SD (UW)**	**χ^2**	**P**	**Composite sample**
Pb	2	0.38 ± 0.10	0.49 ± 0.24	0.92	0.33	0.50
Cd	2	0.42 ± 0.28	0.40 ± 0.16	0.03	0.85	0.43
As	2	0.13 ± 0.04	0.17 ± 0.12	0.41	0.51	0.18
Zn	2	67.32 ± 15.21	75.06 ± 35.67	0.24	0.62	74.1
Cu	2	4.88 ± 1.46	4.93 ± 2.18	0.001	0.96	4.98
Mn	2	49.54 ± 11.95	52.28 ± 23.45	0.06	0.79	54.8
Pb	7	0.44 ± 0.47	0.23 ± 0.06	1.06	0.30	0.27
Cd	7	0.12 ± 0.04	0.07 ± 0.04	2.83	0.10	0.09
As	7	0.33 ± 0.28	0.28 ± 0.06	0.22	0.63	0.33
Zn	7	44.82 ± 12.24	34.82 ± 6.20	2.86	0.09	31.7
Cu	7	8.31 ± 3.69	10.31 ± 2.84	1.08	0.29	10.55
Mn	7	38.22 ± 5.84	43.60 ± 3.93	3.10	0.08	43.3

B) Results of a pilot test to evaluate the percentage of the variance due to differences between and within fields in the concentration of 6 PTE, using five individual soil samples associated the five plants of zacaton (*Sorghum halepense*) in each of two sites (2 and 7) in the agricultural area of San Felipe de Jesús in Sonora, México. Using a generalized mixed model, we partitioned the variance in the concentration of eight PTEs into two components: variation between fields and variation among samples within fields. For most elements, the percentage of the variance due to differences between sites was greater than 90%.

**Element**	**Mean ± SD Site 2**	**Mean ± SD Site 7**	**Percentage of variance due to differences between sites**	**Percentage of variance due to differences within sites**
Pb	167.7 ± 16.1	23.2 ± 0.6	98.75	1.24
Cd	1.4 ± 0.2	0.3 ± 0.1	97.64	2.35
As	11.0 ± 0.8	15.7 ± 0.8	93.98	6.01
Zn	299.0 ± 26.9	72.5 ± 1.8	98.58	1.41
Cu	27.6 ± 2.7	31.8 ± 1.1	64.72	35.27
Mn	936.8 ± 44.85	489.8 ± 8.9	98.96	1.03

Online Resource S3.- Soil properties and concentration of potentially toxic elements among the 26 agricultural fields samples in San Felipe de Jesús, Sonora.

Field	Crop	pH	EC	OC	CEC	As	Cd	Cu	Mn	Pb	Zn	Ca	Fe	K	P	S
1	Peanut	8.58	96.4	0.455	21.25	14.1	0.279	20.4	418	35.3	73.6	1.7	2.4	0.16	0.08	0.01
2	Peanut	8.55	116.9	1.065	16.28	9.57	1.015	22.3	687	117	217	0.5	1.42	0.17	0.04	0.01
3	Peanut	8.55	175.4	0.515	20.96	14.45	0.247	19.9	431	23.3	68.4	1.99	2.52	0.18	0.083	0.01
4	Peanut	8.35	235.0	0.715	14.73	9.07	1.515	28.2	803	168	326	0.47	1.36	0.2	0.038	0.01
5	Peanut	8.18	104.6	0.87	18.96	11.8	1.785	29.2	839	145.5	322	0.51	1.58	0.22	0.049	0.01
6	Pepper	7.98	141.3	1.775	30.875	15.7	5	33.9	757	126.5	649	1.05	1.8	0.23	0.056	0.02
7	Pepper	8.29	198.8	1.23	37.21	15.15	0.544	27.1	760	87.6	122.5	2.24	2.47	0.32	0.095	0.02
8	Maize	8.13	214.3	1.7	19.88	10.1	1.29	30.2	826	136.5	281	0.8	1.52	0.25	0.056	0.02
9	Maize	8.45	156.9	0.84	33.07	17.8	0.461	26.3	655	55.6	102	2.33	2.59	0.27	0.09	0.02
10	Chiltepin	7.96	85.5	1.295	21.05	12.7	6.19	43.7	967	227	781	0.59	1.71	0.22	0.042	0.01
11	Zacaton	8.46	96.46	1.02	20.75	11.05	0.94	25.7	957	110	203	0.66	1.62	0.23	0.05	0.02
12	Zacaton	8.17	318.66	0.94	15.43	8.90	1.33	25.2	920	155.5	289	0.55	1.46	0.22	0.04	0.02
13	Zacaton	8.27	132.53	2.06	40.28	16.45	0.81	35.3	946	182.5	172	2.33	2.27	0.42	0.09	0.03
14	Zacaton	8.29	106.23	0.82	28.15	18.05	0.33	38.2	564	27.2	80.2	2.18	2.49	0.22	0.10	0.01
15	Zacaton	7.99	132.23	1.89	20.92	12.30	1.61	37.4	1010	195	355	0.69	1.67	0.28	0.06	0.02
16	Zacaton	8.05	234.67	2.86	31.30	17.70	0.55	39.2	795	62.2	117	2.02	2.42	0.37	0.12	0.04
17	Zacaton	8.35	156.57	0.94	24.96	15.25	0.29	31.1	475	23.6	72.6	1.98	2.55	0.19	0.09	0.02
18	Zacaton	8.30	141.03	1.52	25.94	19.70	3.66	76.5	1885	367	699	1.11	2.55	0.38	0.06	0.03
19	Zacaton	8.41	85.07	0.59	24.82	16.20	0.28	32.4	546	22.7	78.3	2.20	2.41	0.20	0.09	0.01
20	Zacaton	8.03	211.33	2.36	26.29	18.00	0.66	26.7	720	48.2	146	2.30	2.21	0.38	0.15	0.04
21	Barley	8.34	198.7	1.265	23.2	13.00	1.33	31.8	881	145.5	272	1.03	1.59	0.21	0.06	0.02
22	Barley	8.32	95.7	0.715	11.41	7.23	1.05	20.6	678	121.5	260	0.44	1.13	0.15	0.03	0.01
23	Barley	8.27	162.6	1.43	29.98	10.45	1.38	30.0	802	104.5	275	1.14	1.76	0.20	0.06	0.02
24	Alfalfa	8.52	86.7	1.07	16.22	7.72	0.765	19.2	651	86.8	176	0.57	1.23	0.16	0.038	0.01
25	Alfalfa	8.18	113.6	1.295	16.02	12.95	1.77	45.6	1015	189	392	0.53	1.57	0.2	0.047	0.02
26	Rye Grass-	8.03	212.3	1.135	28.50	18.2	0.381	49.8	632	31.8	84.7	2.42	2.57	0.23	0.1	0.03
	Reference values*					17.6	0.87	21.2	636.8	40.8	80.6	1.6	3.2			

Units. Electrical conductivity (EC in μS/cm), Organic carbon (OC in %), Cation exchange capacity (CEC in cmol/kg), As (mg kg^{-1}), Cd (mg kg^{-1}), Cu (mg kg^{-1}), Mn (mg kg^{-1}), Pb (mg kg^{-1}), Zn (mg kg^{-1}), Ca (%), Fe (ppm), K (%), P (%) and S (%). *Reference values obtained from the geochemistry layer of the Mexican Geological Service of the most important elements in the San Felipe de Jesús area ((https://www.sgm.gob.mx/GeoInfoMexGobMx/#).

Online Resource S4. Pearson correlation plot for soil properties and location with concentration of PTEs in soils and plants. Elements followed by letter s indicate concentration in soil, whereas followed by p indicate concentration in plant. cec: cation exchange capacity; EC: electrical conductivity; Corg: organic carbon; dist_to_mt: distance to mine tailings; dist_to_river: distance to the Sonora River

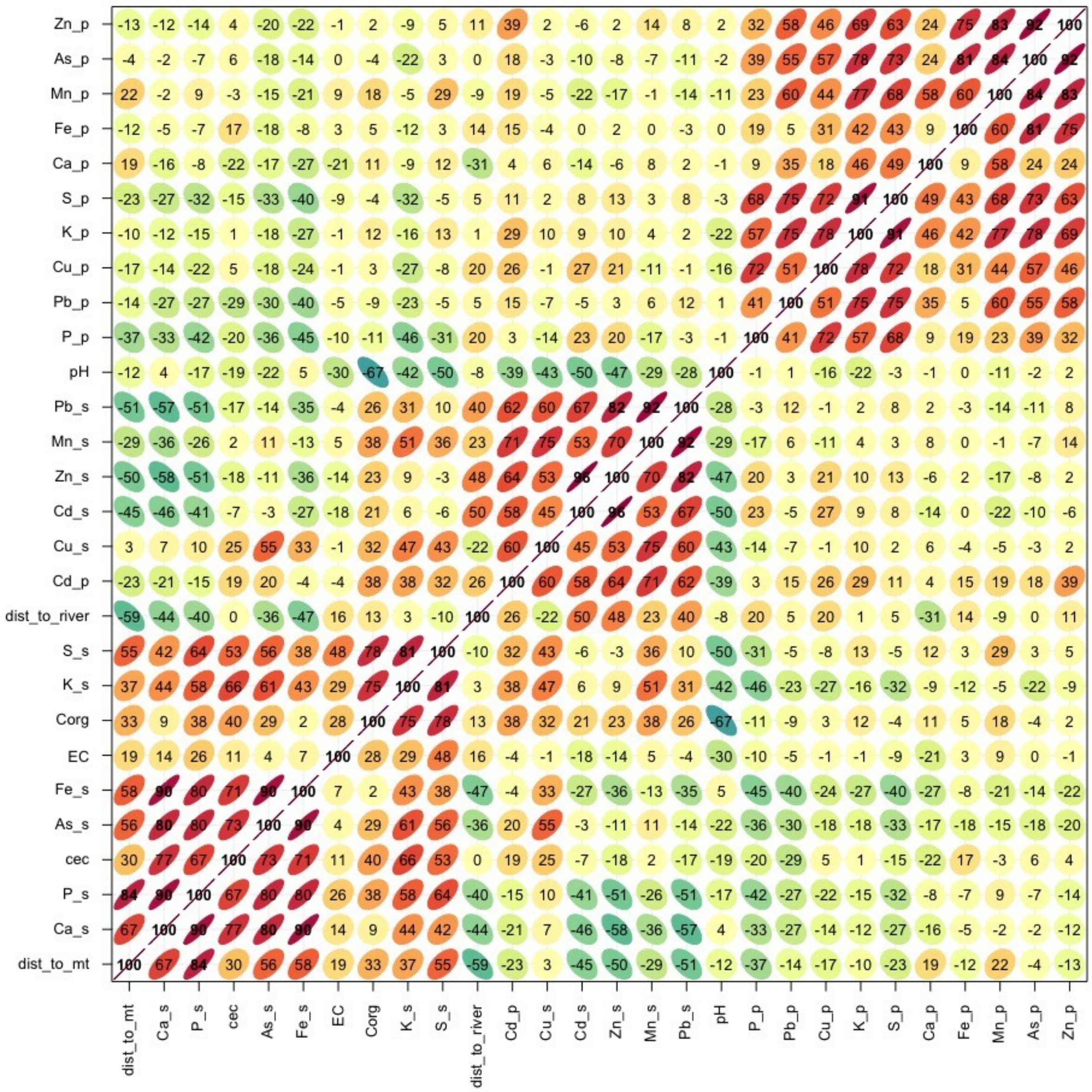

Online Resource S5. Principal component analysis of the concentration of some PTEs in soils from San Felipe

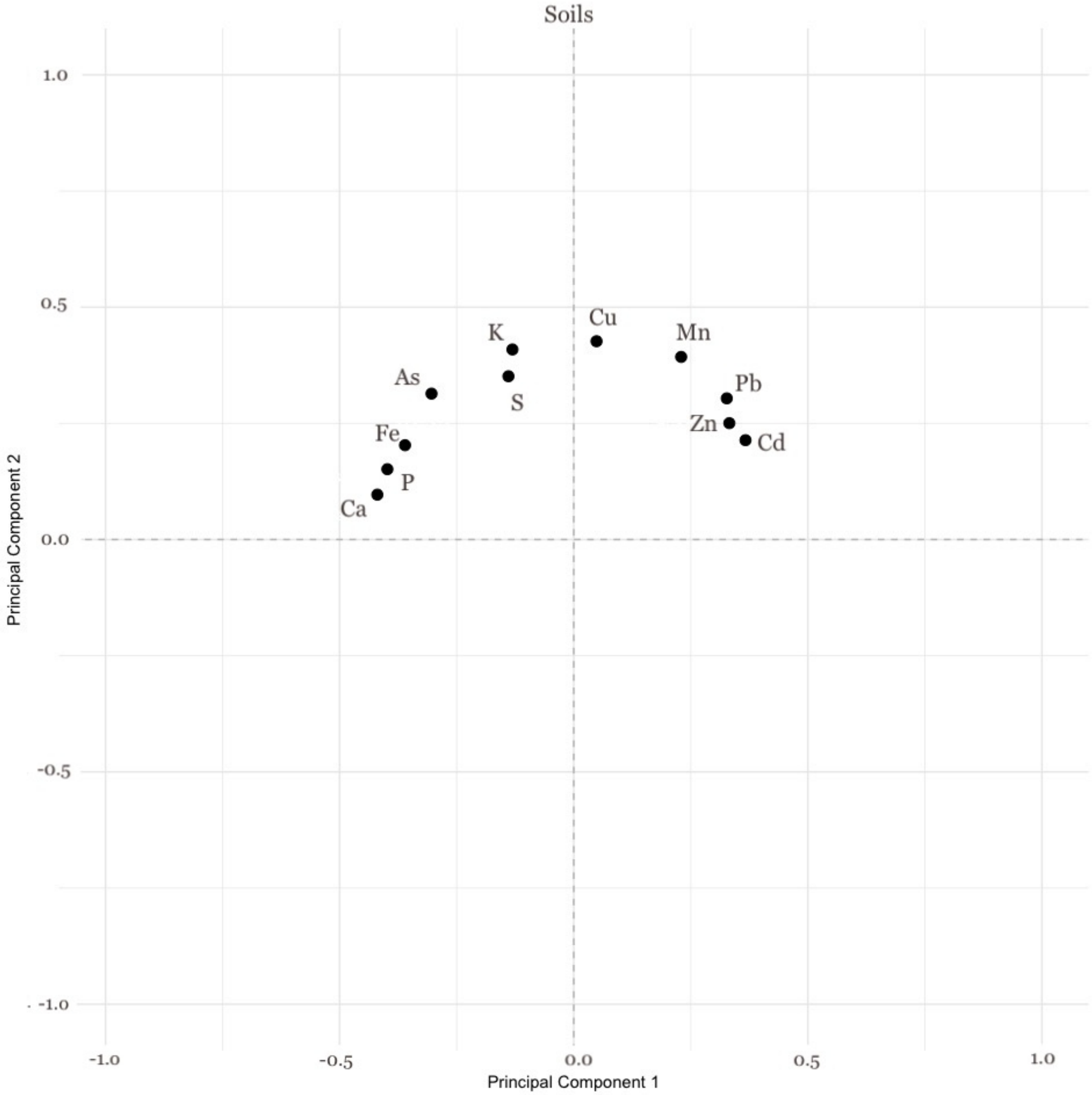

Online Resource S6. Eigenvalues of Principal Component Analysis of the concentration of PTE in soils

Order	Eigenvalue	Percentage of variance	Cumulative percentage
1	5.1652	0.4696	0.4696
2	4.0754	0.3705	0.8401
3	0.8230	0.0748	0.9148
4	0.3953	0.0359	0.9508
5	0.2665	0.02423	0.9750

Online Resource S7. Principal component analysis of the concentration of some PTEs in crops from San Felipe

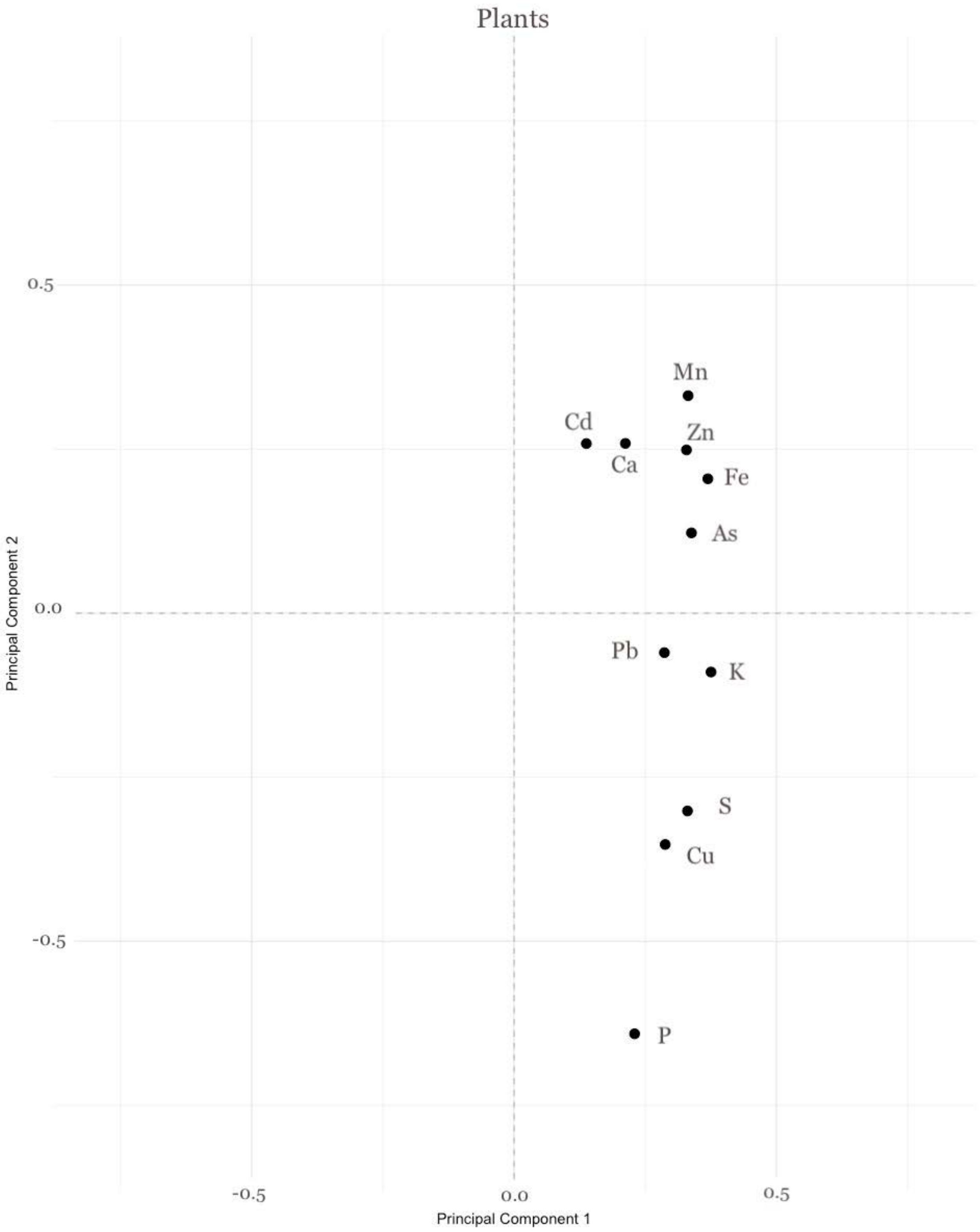

Online Resource S8. Eigenvalues of Principal Component Analysis of the concentration of PTE in plants

Order	Eigenvalue	Percentage of variance	Cumulative percentage
1	6.2375	0.5671	0.5671
2	1.3340	0.1213	0.6883
3	1.0970	0.0997	0.7880
4	0.8910	0.0810	0.8691
5	0.5988	0.0544	0.9235

Online Resource S9. Values of soil indicators and scores of resilience of soil samples from San Felipe used to estimate a resilience index (sensu Song et al. 2022).

Site	pH	SOC	CEC	TFe_2O_3	CaO	Score	Resilience index	Crop	PTE & MTL
1	1	1	4	2	3	11	Moderate	Peanut	PTE < MTL
2	1	2	3	1	2	9	Poor	Peanut	PTE < MTL
3	1	1	4	2	4	12	Moderate	Peanut	PTE < MTL
4	1	1	2	1	2	7	Poor	Peanut	**Cd > MTL**
5	1	1	3	1	2	7	Poor	Peanut	PTE < MTL
6	4	3	4	1	3	15	Moderate	Pepper	**Cd, Pb > MTL**
7	1	2	4	2	4	12	Moderate	Pepper	PTE < MTL
8	1	3	3	1	2	10	Poor	Maize	PTE < MTL
9	1	1	4	2	4	12	Moderate	Maize	PTE < MTL
10	4	2	4	1	2	13	Moderate	Chiltepin	**Cd, Pb > MTL**
11	1	2	4	1	2	10	Poor	Zacaton	PTE < MTL
12	1	1	3	1	2	8	Poor	Zacaton	PTE < MTL
13	1	3	4	1	4	13	Moderate	Zacaton	PTE < MTL
14	1	1	4	2	4	12	Moderate	Zacaton	PTE < MTL
15	4	3	4	1	2	14	Moderate	Zacaton	PTE < MTL
16	1	4	4	2	4	15	Moderate	Zacaton	PTE < MTL
17	1	1	4	2	4	12	Moderate	Zacaton	PTE < MTL
18	1	3	4	2	3	13	Moderate	Zacaton	PTE < MTL
19	1	1	4	2	4	12	Moderate	Zacaton	PTE < MTL
20	1	3	4	1	4	13	Moderate	Zacaton	PTE < MTL
21	1	2	4	1	3	11	Moderate	Barley	**S > MTL**
22	1	1	2	1	2	7	Poor	Barley	**S > MTL**
23	1	2	4	1	3	11	Moderate	Barley	**S, Fe > MTL**
24	1	2	3	1	2	9	Poor	Alfalfa	**S > MTL**
25	1	2	3	1	2	9	Poor	Alfalfa	**S > MTL**
26	1	2	4	2	4	13	Moderate	Rye Grass-barley	**S > MTL**

pH values < 5.5 or > 8 scored as poor (1); between 5.5-6.5 as medium (2); between 6.5-7.5 as good (3), and between 7.5-8 as excellent (4).

Soil Organic Carbon (SOC, %) values <1 scored as poor (1); between 1-1.5 as medium (2); between 1-5-2.5 as good (3) and >2.5 as excellent (4).

Cation Exchange Capacity (CEC, cmol/kg) values <10 scored as poor (1); between 10-15 as medium (2); between 15-20 as good (3) and >20 as excellent (4).

TFe_2O_3 (%) values <3.40 scored as poor (1); between 3.40-4.15 as medium (2); between 4.15-4.60 as good (3) and >4.60 as excellent (4).

CaO (%) values <0.42 scored as poor (1); between 0.42-1.16 as medium (2); between 1.16-2.68 as good (3) and >2.68 as excellent (4).

Score is the sum of the five soil indicators for each soil sample (pH + SOC + CEC + TFe_2O_3 + CaO).

Resilience index: values between 5-10 scored as poor resilience; between 11-16 as moderate resilience and >16 as high resilience.

Crop: crop species grown in each field.

PTE & MTL: indicate whether PTE in the edible part of crop (leaves or fruit/seed) was above (>) or below (<) maximum tolerable levels.

Online Resource S10. Relationships between the concentration of Cd (a) and Pb (b) in leaves and stems (in forage crops) and fruits and seeds (in food crops) with the concentration of Cd and Pb in soil. Pearson correlation coefficients are shown inside each plot

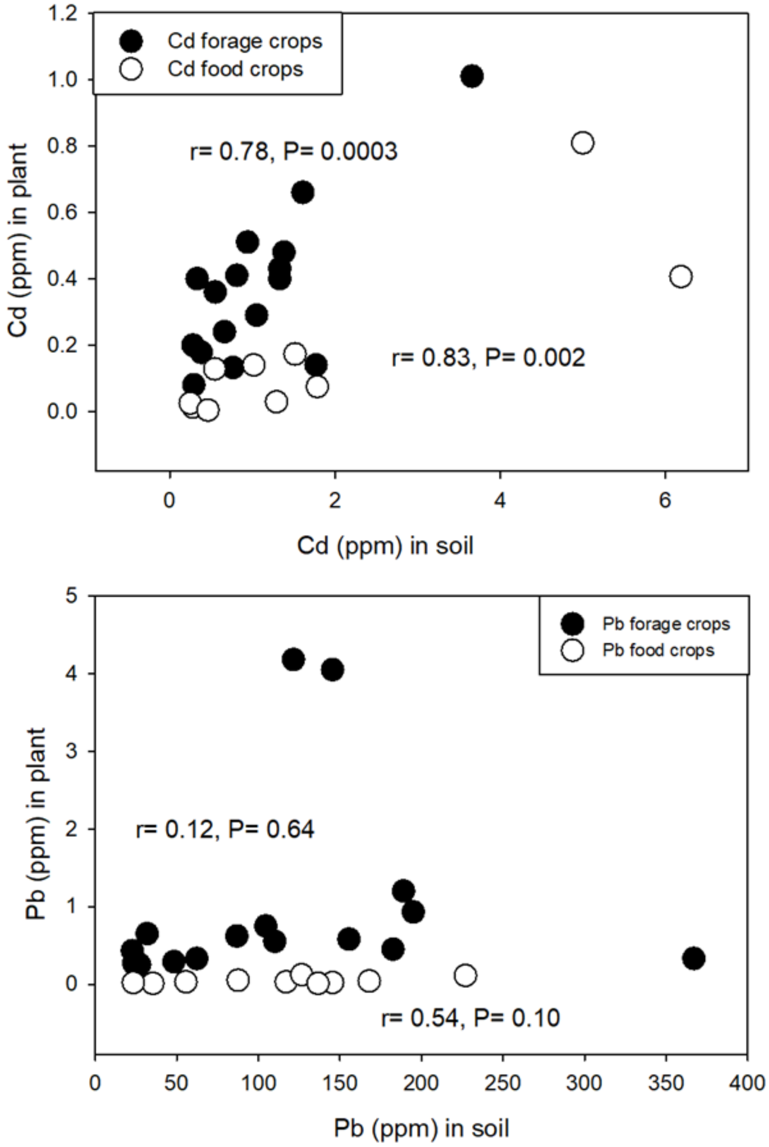

Supplement: Supplementary file 1 — Supplementary file1 (PDF 690 KB) [file 10661_2025_14587_MOESM1_ESM.pdf]
